# Anxiety, Depression, and Post-Traumatic Stress Disorder (PTSD) Symptomatology According to Gender in Health-Care Workers during the COVID-19 Pandemic in Peru Shortened Title: “Psychological Impact of the Pandemic on Women”

**DOI:** 10.3390/ijerph191911957

**Published:** 2022-09-22

**Authors:** Alex Ricardo Martínez Pajuelo, José Eduardo Irrazabal Ramos, Maria Lazo-Porras

**Affiliations:** 1 School of Medicine, Faculty of Health Sciences, Universidad Peruana de Ciencias Aplicadas (UPC), Lima 15067, Peru; 2 CRONICAS Centre of Excellence in Chronic Diseases, Universidad Peruana Cayetano Heredia, Lima 15066, Peru; 3 Division of Tropical and Humanitarian Medicine, University of Geneva and Geneva University Hospitals, 1205 Geneva, Switzerland

**Keywords:** coronavirus infections, Post-Traumatic Stress Disorder, anxiety disorder, depression, mental health

## Abstract

Objective: The current study will evaluate the association that the COVID-19 pandemic has had with health-care workers and identify the factors that influenced the female gender being more affected. Methods: This is a cross-sectional study conducted in two hospitals in Arequipa (a Peruvian city). The participants were health-care workers. We applied a questionnaire with sociodemographic information and three scales: the Generalized Anxiety Disorder-7, the Patient Health Questionnaire-9, and the Primary Care Post-Traumatic Stress Disorder (PTSD) Screen for DSM-5. The main outcomes were anxiety, depression, and PTSD scores. The exposure of interest was gender. The scores of the scales were estimated by medians and percentiles 25–75 (p25–p75), and we used linear regression to estimate the crude and adjusted coefficients and their respective confidence intervals at 95% (CI 95%). Results: There were 109 participants, and 43.1% were women. The anxiety, depression, and PTSD median (p25–p75) scores in the study population were 6 (2–11), 6 (2–10), and 1 (0–3), respectively. The adjusted analysis showed that the female sex had 4.48 (CI 95% 2.95–6.00), 4.50 (CI 95% 2.39–6.62), and 1.13 (CI 95% 0.50–1.76) higher points on average for the scales of anxiety, depression, and PTSD symptoms in comparison to males, respectively. Conclusions: Female health-care workers showed increased scores of mental health issues in comparison to male health-care workers.

## 1. Introduction

When the year 2019 was ending, a new SARS variant was identified, SARS-CoV-2, in Wuhan (China) [1]. On 11 March 2020, the World Health Organization (WHO) declared the COVID-19 outbreak a pandemic. The Peruvian government implemented different safety and containment measures, such as social distancing and a State of Emergency [2]. These measures affected the mental health of the population [3].

Mental health issues have been rising in the health-care workers population throughout their fight against the COVID-19 pandemic; health-care workers and hospital workers are a group highly exposed to anxiety, depression, stress, and other mental health problems [4]. It is known that some contributing factors that increase these issues are the high rate of transmission of COVID-19, the high mortality rate of this disease, and the physical and mental exhaustion that occurs from caring for the sick [4,5].

Mental health issues have prevailed in health-care workers, such as burnout syndrome and depression [6,7]. This population has been extremely vulnerable to mental health symptoms during the COVID-19 pandemic. Moreover, the prevalence of psychiatric symptoms (anxiety and depression) and risky behaviours (alcohol consumption, smoking, and social isolation) increased in health-care workers during the COVID-19 pandemic [8,9]. Additionally, there are studies confirming that frontline health-care workers during the COVID-19 pandemic are predisposed to developing PTSD, an aerosol disease defined as a clinical picture produced by exposure to traumatic and stressful events (fear of contagion and/or infecting family members, death of family members and/or friends, or collapse of the health system). The development of PTSD depends not only on the type of event exposure but also on its intensity and frequency [10]. However, the impact on the mental health of health-care workers differs in regard to their gender. A study in Paraguay showed female health-care workers presented more severe symptoms of depression, anxiety, stress, and fatigue in comparison to their male counterparts [9]. Studies in Brazil, China, and Spain identified higher anxiety, depression, and stress symptoms in women, health science students, and/or having physical symptoms or previous health problems [11,12]. Additionally, a systematic review concluded that women, including nurses, are at a high risk of developing PTSD during the COVID-19 pandemic compared to men [13,14].

There are existing factors that can predispose women toward a higher frequency of mental illnesses, such as social roles and the influence of the reproductive cycle. In one hand, the sociocultural factor related to gender and the role that being a woman plays in different societies and, on the other hand, the hormonal changes from the reproductive cycle influence their state of mind [15]. Premenstrual syndrome (PMS) is an illness that can occur during the menstrual cycle, with a prevalence of 5–8%, classified under psychiatric illnesses and that results in irritability, depression, anxiety, and insomnia. These illnesses can cause a depressive episode or worsen the depressive symptomatology [16].

The previously mentioned studies evidenced an increase in the psychiatric symptomatology in health professionals, gender being one of the associated variables. Nevertheless, previous studies have been exploratory and have not been able to approach with enough depth the evaluation of gender as a risk factor for some mental health problems during the COVID-19 pandemic. The association between mental health issues and gender during the pandemic has been studied in a limited way and lacks the consideration of the confounding factors of this relationship.

This study will evaluate the association that the COVID-19 pandemic has had with health-care workers and identify the factors that influenced the female sex being more affected. This information will allow researchers and decision makers to establish mental health strategies based on evidence and focus on vulnerable subgroups. Furthermore, it is important to highlight that Peru has been considered one of the most affected countries regarding the COVID-19 pandemic [17,18]. Thus, the aim of the study is to compare the anxiety, depression, and PTSD symptomatology regarding the sex (female vs. male) of the health-care workers in two hospitals located in Arequipa, a Peruvian city. Additionally, we aimed to identify the anxiety, depression, and PTSD-associated factors and describe the frequency of these mental health problems in this population.

## 2. Methods and Materials

### 2.1. Study Design and Context

This was a cross-sectional study. A survey was applied from May to July (weeks 19–31) of 2021 at two reference hospitals located in Arequipa, a region in the highlands of Peru. One hospital provides care to the lower-income population in the region, and the other hospital provides care to the members of the police and their families.

The second peak (2nd wave) of infections in the COVID-19 pandemic was experienced in the city of Arequipa between weeks 17 and 31 [19]. In addition, it is important to mention that the vaccination of health-care workers began in the city of Arequipa on 11 March 2021 [20], ending at approximately week 20 [21].

### 2.2. Population

**Figure 1:**
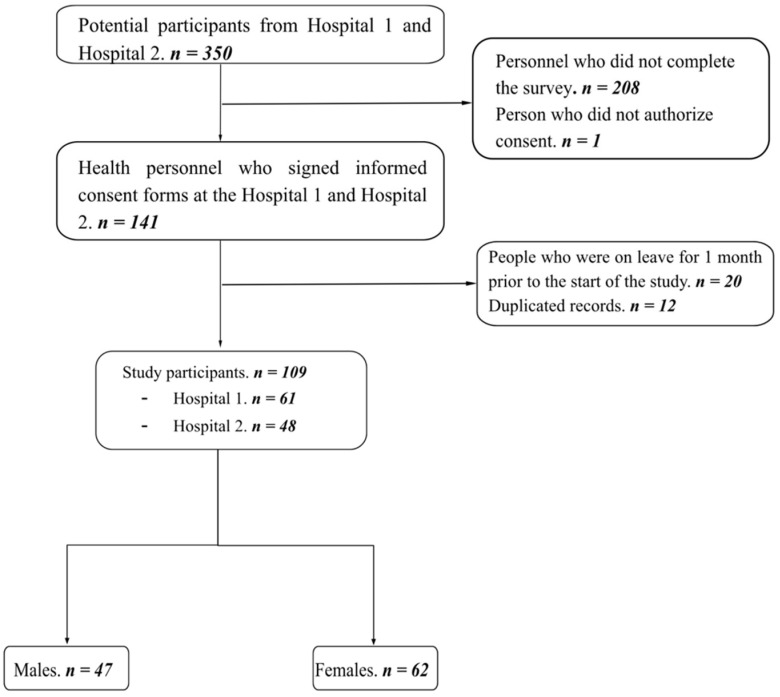
Flowchart.

The study included medical, nursing, and technical staff working during the COVID-19 pandemic in the above-mentioned hospitals. The population was sampled by convenience in a non-probabilistic way. The inclusion criteria were being health-care workers who worked in person during the pandemic between April 2021 and July 2021. Additionally, only participants who completed the entire survey were included to ensure adequate sensitivity and specificity of the tests used to assess anxiety, depression, and PTSD symptoms. In terms of the exclusion criteria, staff who were on leave for a period of more than 2 months between September 2020 and May 2021 were not considered. More information is available in Figure 1.

The outcome variables were anxiety scores according to the Generalized Anxiety Disorder-7 (GAD-7) scale, the depression scores determined with the Patient Health Questionnaire-9 (PHQ-9) scale, and the PTSD scores measured with the Primary Care Post-Traumatic Stress Disorder Screen for DSM-5 (PC-PTSD-5), while the independent variable was gender/sex (Male/Female).

Additionally, using the previously mentioned scales, anxiety, depression, and PTSD frequency were estimated with the cut off points of ≥10, ≥10, and ≥3, respectively [22,23,24,25].

The assessed covariates were: age (≤45 or >45); marital status (single, married/cohabitant, or divorced); number of children (no children, 1 to 2 children, or 3 to 4 children); profession (physician, nurse, or technical staff); hospital (hospital 1 or hospital 2); area (COVID-19 area, non-COVID-19 area, or both); service where they worked (emergency, triage COVID-19, hospitalization non-COVID-19, hospitalization COVID-19, or outpatient consultation); hours of work (≥150 h/month or <150 h/month); medical history of comorbidities (Yes or No); and loss of family member/close friend (Yes or No).

### 2.3. Procedures

An electronic questionnaire was designed that contained the informed consent, sociodemographic information, and background of the participants. Initially, the link to the survey was sent via phone applications. Later in the process, a printed copy was hand-delivered to the health-care workers to be filled out.

The GAD-7 scale is a questionnaire of seven questions with four types of answers (0 = never, 1 = less than half the time, 2 = more than half the time, and 3 = almost always) with a maximum score of 21. It evaluates symptoms of anxiety from the past two weeks, and it was validated by a U.S.A. study; the alpha’s Cronbach was 0.92 [22]. The PHQ-9 scale consists of nine questions with four types of answers (0 = never, 1 = sometimes, 2 = most of the time, and 3 = almost always) with a maximum score of 27; the alpha’s Cronbach was 0.84. It evaluates depressive symptoms in the previous weeks, and it was validated by studies performed in Chile [23,24]. Additionally, the PC-PTSD-5 scale included a screening question about a specific traumatic event. In this case, the participants were asked if they considered their work in a health-care facility during the COVID-19 pandemic a more stressful event in comparison to other events. This test has five questions with two types of answers (Yes/No), with a maximum score of 5. It evaluates PTSD symptoms in the last month, and it was validated by studies in the USA.; the alpha’s Cronbach was 0.83 [25].

### 2.4. Power Estimation

The power was estimated using OpenEpi, and the scores were from PHQ-9 in a Greek study performed on health professionals. The average score of depressive symptoms in women was 14.74 (SD 4.31) and men was 12.49 (SD 2.82) [26]. The difference between both groups was 2.5. We estimated the population based on the number of health-care workers that work in both hospitals (350 participants). Assuming 175 are men and 175 are women and an acceptance rate of 25%, the power was 81.7%.

### 2.5. Analysis Plan

For the numeric variables, Shapiro Wilk was used to evaluate the normal nature of the scores and, thus, present the information through means and standard deviations or medians and percentiles (p25–p75). For the categorical variables, we used the frequency and percentages. The depression, anxiety, and PTSD frequency were also described in the sample. In all the tests we evaluated, the assumptions were based on the test used. For the bivariate analysis, we used the *t-*test to evaluate the outcome variables (C. anxiety, C. depression, and C. PTSD) with the dichotomous categorical covariables when the normality criteria were fulfilled, whereas, when this criterion was not fulfilled, the Mann–Whitney test was used. For the categorical variables with three or more categories, we used the ANOVA test. In the multivariate analysis, we applied simple linear regression to estimate the differences between averages (Coef) of the reference and one or more categories in the anxiety, depression, and PTSD scores, as well as its 95% confidence intervals (95% CI). We performed a crude and adjusted analysis, and the criteria to adjust were based on the theory and included in the model those considered confounding variables. We evaluated the collinearity of the confounding variables in the model. The statistical significance used was *p* < 0.05.

### 2.6. Ethics

The study was approved by the Ethics Committee of the Universidad Peruana de Ciencias Aplicadas, and approval from the two hospitals was obtained. One of the hospitals did not give us permission to recruit nurse participants; however, we did manage to obtain permission over all the other areas. Moreover, the informed consent in the virtual surveys was obtained through a Google Forms survey, but with some participants, a consent form was printed and signed. We kept the confidentiality of all the participant’s names using codes. The ethical principles of the participants were respected in accordance with the Helsinki Declaration [27].

## 3. Results

### 3.1. Study Population Characteristics

A total of 109 participants were included in Hospital 1 (61 participants) and in Hospital 2 (48 participants); flowchart 1 shows the inclusion of the participants. The study population was 56.9% women and 43.1% men. The mean age was 44.8 (SD 9.2), the youngest participant was 28 years old, and the oldest was 69 years old. The frequency of married/cohabitants participants was 60.6%, single 28.4%, and 11% divorced. Over half (57.4%) of the participants had between one and two children, 18.5% had three to four, and 24.1% had no children. The anxiety (C. anxiety), depression (C. depression), and PTSD (C. TEPT) scores had a median and p25–p75 of 6 (2–11), 6 (2–10), and 1 (0–3), respectively. The frequency of anxiety was 32.11%, depression was 30.28%, and PTSD was 31.19% in Table 1.

### 3.2. Potential Factors Associated to Anxiety, Depression, and PTSD

The results showed that the female sex had higher anxiety, depression, and PTSD mean scores compared to males: 8.7 vs. 3.7, 8.7 vs. 3.8 and 2.0 vs. 1.0, respectively. Additionally, differences were found in the anxiety scores between hospitals: hospital 1 and hospital 2 had a mean of 7.7 vs. 5.1 (*p* = 0.002). On the other hand, in the case of depression, the factor that showed an association was working hours, in which the category <150 h/month vs. ≥150 h/month had mean scores of 8.1 vs. 4.2 (*p* < 0.001). In the case of PTSD, the factor that showed association was working hours, the category of <150 h/month vs. ≥150 h/month with mean scores of 2.0 vs. 1.0 (*p* = 0.004). Variables that showed no association for any of the disorders assessed were age, number of children, profession, medical history, and loss of family member/close friend. More information on anxiety, depression, and PTSD scores with potential associated factors is available in Table 2.

### 3.3. Factors Independently Associated with Anxiety, Depression, and PTSD

Firstly, in the crude analysis, it is seen that the female sex has, on average, higher anxiety, depression, and PTSD symptomatology scores compared to males (coef = 4.96, 95% CI 3.54–6.38), (coef = 4.87, 95% CI 3.0–6.75), (coef = 1.04, 95% CI 0.46–1.61), respectively. In the case of marital status, married/cohabitant people have 1.84 points more on average of anxiety symptoms compared to single participants (coef = 1.84, 95% CI 0.02–3.66). However, in the assessment of depression, it was found that divorced people had lower scores than single people (coef = −4.01, 95% CI −7.59 to −0.43). Additionally, it was found that people who work fewer hours (<150 h/month) have higher symptomatology of anxiety, depression, and PTSD (coef = 3.29, 95% CI 1.62–4.97), (coef = 3.93, 95% CI 1.83–6.03), and (coef = 0.92, 95% CI 0.30–1.54), respectively.

Finally, in the adjusted analysis, it was found that the female sex, when adjusted for confounding variables, had 4.48, 4.50, and 1.13 higher mean scores of anxiety, depression, and PTSD symptoms than males, respectively. We tested for collinearity and found no evidence. More information related to the other variables can be found in Table 3.

## 4. Discussion

The principal finding was the significant differences in the average scores of the anxiety, depression, and PTSD symptoms in the female sex in comparison to males. Furthermore, in the crude models, the group with higher anxiety symptoms was those married/cohabitants in comparison to the singles group. On the other hand, it was found that depressive and PTSD scores were smaller in divorced people compared to single people. Another finding was that the health-care workers that worked fewer hours (<150 h/month) were the ones that obtained higher scores in anxiety, depression, and PTSD symptoms in the crude analysis.

The COVID-19 pandemic has increased the anxiety, depression, distress, and insomnia symptoms levels in people compared to previous years [7]. The health-care workers population has an increased risk in developing mental health issues because of the challenges they face [28]. In a study from China, it was found that the prevalence of anxiety and depressive symptoms was higher in health-care workers in comparison to the general population [29,30]. Additionally, other risk factors can be associated with mental illnesses in the general population, such as being a woman, being a nurse, suffering from risk comorbidities, social isolation, and more time spent watching COVID-19-related news [31].

We identified that female health-care workers present more anxiety, depression, and PTSD symptoms in comparison to male health-care workers, as many different studies have shown. A systematic review of health professionals during the COVID-19 pandemic identified studies from Italy, Spain, Turkey, the UK, Romania, Serbia, and the USA.; these studies showed that stress, anxiety, depression, sleep deprivation, and burnout symptoms were higher in women in comparison to men, particularly in nurses [32]. Additionally, we found that the anxiety rate and anxiety scores in the female health-care workers were much higher than in the male-care workers (43.13 ± 11.12) vs. (39.14 ± 9.01), and the same occurred when evaluating the PTSD levels, where they found that women scored higher than men [4]. In relation to their profession, nurses had higher anxiety, depression, and PTSD symptomatology scores in comparison to the rest of the health-care workers [33], similar results were seen in the present study but only in the crude analysis. Some factors associated with the nurse profession are taking care of affected patients, having less experience, or having a half-time job [28,32,33,34,35,36]. Additionally, one odd finding was that health-care workers who worked fewer hours (<150 h/month) obtained higher scores in symptoms of anxiety, depression, and PTSD in the crude analysis. This was a finding in the crude analysis but not in the adjusted one, and the effect can be caused by a confounding factor, e.g., there is evidence that women in the medical field work fewer hours than men [37]; similarly, another study found that a women work fewer hours for each additional kid [38]. Given that this is a cross-sectional study, it does not evaluate causality, and therefore, it is possible that the health-care workers with anxiety, depression, and PTSD symptoms have decided to reduce their working hours or that the hospital itself has brought them the chance to reduce their working days [39]. Another explanation is that not only the COVID-19 pandemic affects health-care workers psychologically, but also, the measures implemented by the government (quarantine and social isolation) aggravated them even more, so it is possible that these can explain that those who work less hours and spend more time at home have more symptoms of anxiety, depression, and PTSD [40].

According to the National Institute of Mental Health (NIMH), PTSD is a “disorder that develops in people who have experienced a shocking, frightening or dangerous event. It can arise as a result of a single isolated event or as a product of more chronic or repeated traumatic experiences.” Health-care workers during the COVID-19 pandemic experienced many repeated traumatic events, such as fear of becoming infected or contagious, fear of dying or seeing family members and colleagues die, collapse of the health-care system, etc., so these events could be classified as traumatic events with a probable diagnosis of PTSD [41]. Additionally, in past pandemics, the exposure of health-care workers to patients infected with the SARS virus was considered a traumatic, life-threatening event [42].

However, there have been previous studies in the general population that mentioned the bias in PTSD screening in relation to the pandemic by COVID-19, because it could incur in an overestimation of the prevalence of PTSD. The identification of the specific traumatic event of criterion A would not be fulfilled, since there are several events that could influence the identification of the event, such as family (domestic abuse during quarantine during the pandemic) and hospital (fear of infection and deaths of family members and colleagues), among other traumatic experiences. Thus, the vagueness of a traumatic event encompassing the COVID-19 pandemic would make it difficult to differentiate the specific traumatic event for the diagnosis of PTSD [40,42,43,44].

### 4.1. Relevance for the Mental Health of Health-Care Workers

Our study has found high anxiety, depression, and PTSD symptoms scores and frequencies in health-care workers from Arequipa, besides a higher score of symptoms in the female vs. the male sex. This scenario was found even though, from April 2020, a new mental health guide was approved (Guía Técnica para el Cuidado de la Salud Mental del Personal de salud en el contexto del COVID-19) in Peru and also counts access to the 113 hotline to provide support to health-care workers [45]. Despite this guide, our findings show the pending work to protect health-care workers according to gender differences and promote in them strategies to recover or emotional well-being maintenance.

### 4.2. Limitations and Strengths

The lower acceptance rate for health-care workers can lead to the overestimation and underestimation of results; this is why we used both physical and virtual tools to obtain a big participant pool. It can generate an underestimation of the symptoms given the survey was done after the health-care workers received their first round of vaccines, and the participants could have felt more protected and secure; however, this contrasts with the context, as the city found itself in the midst of the second wave, and this could have exacerbated their symptoms. On the other hand, one of the hospitals did not give us authorization to conduct the survey in the nurse department and did not fill out the surveys. Related to the internal validity, there may be an information bias, because it is possible that the participants did not remember presenting symptoms during the last month; this period of time is taken as a reference due to the nature of the data collection tools. Additionally, there is controversy about considering the COVID-19 pandemic as a traumatic event for health-care workers. It is possible that we overestimated the prevalence of PTSD in our studied population.

Other factors that can have an impact on the study are the absence of internet on their phone and/or damaging the surveys when they were printed or them being lost at the moment of their return. We did not collect information about the dedication to household activities and the attention given to their kids or other family members because of an inadequate filling out of the answers.

The strengths we considered in our study gave more importance to mental health in health-care workers, repowering various factors of each hospital, such as infrastructure, work schedules, assessment, and tracing given by the hospitals. Different studies show women as one of the most affected groups by this pandemic in relation to the psychological impact [46]. We have to consider the effect of mental health problems on the health-care workers as increments in human errors; faulty decision making; lack of compromise; a decrease in motivation, tension, and discussions with colleagues; etc. Furthermore, it is important, because Peru does not have strategies to avoid mental illness in health-care workers.

It is recommended to search for strategies to collect information about mental health in health-care workers. Furthermore, it would be important to perform longitudinal studies for the follow-up and control of the evolution of anxiety, depression, and PTSD symptoms in regard to gender. Similarly, we recommend that, during the pandemic, there is an implementation of evaluations and interventions for the diagnosis and management of mental health issues in health-care workers considering their gender.

## 5. Conclusions

In different studies about mental health during the COVID-19 pandemic, there has been evidence of a higher symptomatology of mental illness in health-care workers. However, they have not yet evaluated the external factors that could potentially make a difference in the symptomatology scores in a population. In our study, we showed an adjusted analysis of how the sex of health-care workers can change the average score of their anxiety, depression, and PTSD symptomatology. Therefore, we need to implement strategies to maintain and improve the mental health of the health-care workers, not only during emergency crisis situations and considering gender in the development of these interventions.

## Figures and Tables

**Table 1:** Characteristics of the participants.

Variable	N = 109	
**Age (years) *n* (%)**		
≤45	54	49.5%
>45	55	50.5%
**Sex, *n* (%)**		
Female	62	56.9%
Male	47	43.1%
**Marital Status, *n* (%)**		
Single	31	28.4%
Married/cohabitant	66	60.6%
Divorced	12	11.0%
**Number of Children, *n* (%)**		
No children	26	24.1%
1–2 children	62	57.4%
3–4 children	20	18.5%
**Profession, *n* (%)**		
Nurse	35	32.4%
Technical staff	29	26.9%
Physician	44	40.7%
**Labor hospital, *n* (%)**		
Hospital 1	61	56.0%
Hospital 2	48	44.0%
**Occupation, *n* (%)**		
COVID-19 area	32	29.6%
Non-COVID-19 area	20	18.5%
Both	56	51.9%
**Service where you work, *n* (%)**		
Emergency	10	9.4%
Triage COVID-19	6	5.6%
Non-COVID-19 Hospitalization	37	35.0%
COVID-19 Hospitalization	36	34.0%
Consultation	17	16.0%
**Working hours, *n* (%)**		
<150 h	64	63.4%
≥150 h	37	36.6%
**Pathological history, *n* (%)**		
Yes	16	14.7%
No	93	85.3%
**Loss of family member/close friend, *n* (%)**		
Yes	61	56.0%
No	48	44.0%
**Anxiety score, median (p25–p75)**	6	(2–11)
**Depression score, median (p25–p75)**	6	(2–10)
**PTSD score, median (p25–p75)**	1	(0–3)

PTSD: Post-Traumatic Stress Disorder.

**Table 2:** Bivariate analysis with respect to the anxiety, depression, and PTSD scores obtained.

	C. Anxiety	*p*	C. Depression	*p*	C. PTSD	*p*
Age (**), median (DE)						
≤45	6.5 (4.4)	0.923	7.0 (6.0)	0.446	1.7 (1.7)	0.307
>45	6.6 (4.5)		6.2 (4.8)		1.4 (1.3)	
Sex (**), median (DE)						
Female	8.7 (3.5)	**<0.001**	8.7 (4.8)	**<0.001**	2.0 (1.5)	**<0.001**
Male	3.7 (4.0)		3.8 (5.0)		1.0 (1.4)	
Marital Status (***), median (DE)						
Single	5.7 (4.3)	**0.002**	6.7 (6.0)	**0.030**	1.8 (1.8)	0.088
Married/cohabitant	7.5 (4.4)		7.2 (5.2)		1.6 (1.4)	
Divorced	3 (2.8)		2.7 (4.0)		0.6 (1.2)	
Number of Children (***), median (DE)						
No children	5.4 (4.6)	0.059	6.5 (6.2)	0.116	1.8 (1.8)	0.742
1–2 children	6.4 (4.2)		5.9 (5.1)		1.5 (1.5)	
3–4 children	8.5 (4.6)		8.8 (5.3)		1.5 (1.1)	
Profession (***), median (DE)						
Nurse	7.7 (4.5)	0.100	8.1 (6.1)	0.125	2.1 (1.4)	0.051
Technical staff	6.7 (5.0)		5.6 (4.4)		1.3 (1.3)	
Physician	5.5 (4.0)		6.0 (5.4)		1.3 (1.6)	
Labor hospital (**), median (DE)						
1	7.7 (4.3)	**0.002**	7.0 (4.6)	0.361	1.5 (1.2)	0.467
2	5.1 (4.2)		6.0 (6.3)		1.7 (1.8)	
Occupation (***), median (DE)						
COVID-19 area	4.8 (3.9)	**0.020**	5.4 (5.7)	0.330	1.7 (1.6)	0.837
Non-COVID-19 area	6.4 (4.5)		7.6 (6.2)		1.7 (1.8)	
Both	7.6 (4.5)		6.9 (5.0)		1.5 (1.4)	
Service where you work (***), median (DE)						
Emergency	6.9 (4.7)	0.150	5.7 (4.0)	0.570	0.8 (1.0)	0.121
Triage COVID-19	4.7 (4.4)		4.7 (6.4)		1.5 (1.0)	
Non-COVID-19 Hospitalization	7.9 (4.3)		7.8 (5.6)		1.9 (1.6)	
COVID-19 Hospitalization	5.5 (4.4)		6.3 (6.0)		1.7 (1.7)	
Consultation	6.9 (4.0)		6.1 (4.7)		0.9 (1.0)	
Working hours (**), median (DE)						
<150 h	7.9 (4.5)	**<0.001**	8.1 (5.5)	**<0.001**	2.0 (1.5)	**0.004**
≥150 h	4.6 (3.4)		4.2 (4.3)		1.0 (1.4)	
**Pathological history (+), median (p25–p75)**						
Yes	8 (2.5–11)	0.454	9 (4.5–13.5)	0.056	3 (0–4)	0.097
No	6 (2–11)		5 (2–10)		1 (0–2)	
Loss of family member/close friend (**), median (DE)						
Yes	6.5 (4.8)	0.900	6.7 (6.0)	0.836	1.7 (1.7)	0.403
No	6.6 (4.0)		6.4 (4.7)		1.4 (1.3)	

Anxiety: Anxiety score, C. Depression: Depression score, C. PTSD: Post-Traumatic Stress Disorder, and SD: Standard deviation. (**) Student’s *t*-test, (***) ANOVA, and (+) Mann–Whitney *U* test. *p* values in bold are significant values.

**Table 3:** Potential associated factors with anxiety, depression, and PTSD.

	C. Anxiety		C. Depression		C. PTSD	
**Variable**	**Crude**	Adjusted *	**Crude**	Adjusted *	**Crude**	Adjusted *
	**Coef (CI 95%)**	**Coef (CI 95%)**	**Coef (CI 95%)**	**Coef (CI 95%)**	**Coef CI 95%**	**Coef CI 95%**
**Age**						
≤45	Ref	Ref	Ref	Ref	Ref	Ref
>45	0.08 (−1.60–1.77)	−1.23 (−2.77–0.31)	−0.80 (−2.87–1.27)	−1.54 (−3.68–0.60)	−0.31 (−0.91–0.29)	−0.40 (−1.05–0.25)
**Sex**						
Female	Ref	Ref	Ref	Ref	Ref	Ref
Male	**4.96 (3.54–6.38)**	**4.48 (2.95–6.00)**	**4.87 (3.0–6.75)**	**4.50 (2.39–6.62)**	**1.04 (0.46–1.61)**	**1.13 (0.50–1.76)**
**Marital Status**						
Single	Ref	Ref	Ref	Ref	Ref	Ref
Married/cohabitant	**1.84 (0.02–3.66)**	1.35 (−0.93–3.63)	0.53 (−1.76–2.83)	1.11 (−2.06–4.27)	−0.14 (−0.82–0.54)	0.33 (−0.61–1.27)
Divorced	−2.71 (−5.55–0.13)	−0.56 (−3.24–2.12)	**−4.01 (−7.59–−0.43)**	−1.58 (−5.30–2.14)	**−1.15 (−2.21–−0.09)**	−0.63 (−1.74–0.48)
**Number of Children**						
No children	Ref	Ref	Ref	Ref	Ref	Ref
1 to 2 children	0.98 (−1.04–3.00)	0.27 (−2.05–2.59)	−0.65 (−3.15–1.85)	−0.16 (−3.38–3.05)	−0.28 (−1.02–0.46)	−0.07 (−1.03–0.88)
3 to 4 children	**3.08 (0.50–5.65)**	1.41 (−1.65–4.48)	2.26 (−0.92–5.45)	2.30 (−1.96–6.55)	−0.26 (−1.23–0.71)	−0.27 (−1.58–1.04)
**Profession**						
Nurse	Ref	Ref	Ref	Ref	Ref	Ref
Technical staff	−1.03 (−3.22–1.16)	0.40 (−1.76–2.56)	−2.50 (−5.18–0.20)	−0.65 (−3.65–2.35)	−0.78 (−1.58–0.02)	0.13 (−0.78–1.05)
Physician	**−2.16 (−4.14–−0.19)**	−0.26 (−2.05–1.54)	−2.11 (−4.54–0.31)	−0.79 (−3.28–1.70)	**−0.78 (−1.46–−0.09)**	−0.42 (−1.16–0.32)
**Labor hospital**						
Hospital 1	Ref	Ref	Ref	Ref	Ref	Ref
Hospital 2	**−2.57 (−4.20–−0.94)**	−0.27 (−2.66–2.11)	−0.96 (−3.04–1.12)	1.18 (−2.12–4.49)	0.22 (−0.38–0.83)	0.68 (−0.30–1.66)
**Occupation**						
COVID-19 area	Ref	Ref	Ref	Ref	Ref	Ref
Non-COVID-19 area	1.56 (−0.89–4.00)	0.86 (−1.70–3.41)	2.16 (−0.92–5.24)	1.45 (−2.08–4.99)	0.07 (−0.90–1.03)	−0.22 (−1.27–0.82)
Both	**2.73 (0.83–4.63)**	1.35 (−0.42–3.12)	1.44 (−0.96–3.83)	0.31 (−2.15–2.77)	−0.16 (−0.85–0.54)	−0.37 (−1.10–0.36)
**Service where you work**						
Emergency	Ref	-	Ref	-	Ref	-
Triage COVID-19	−2.23 (−6.70–2.23)	-	−1.03 (−6.69–4.62)	-	0.72 (−0.85–2.29)	-
Non-COVID-19 Hospitalization	1.02 (−2.06–4.10)	-	2.08 (−1.82–5.99)	-	1.14 (0.02–2.25)	-
COVID-19 Hospitalization	−1.4 (−4.49–1.69)	-	0.58 (−3.34–4.49)	-	0.96 (−0.16–2.07)	-
Consultation	−0.02 (−3.46–3.43)	-	0.42 (−3.95–4.78)	-	0.16 (−1.10–1.41)	-
**Working hours**						
≥150 h	Ref	Ref	Ref	Ref	Ref	Ref
<150 h	**3.29 (1.62–4.97)**	1.60 (−0.09–3.29)	**3.93 (1.83–6.03)**	2.14 (−0.20–4.48)	**0.92 (0.30–1.54)**	**0.80 (0.10–1.49)**
**Pathological history**						
Yes	Ref	Ref	Ref	Ref	Ref	Ref
No	−0.77 (−3.16–1.60)	−0.37 (−2.43–1.69)	**−3.08 (−5.95–−0.21)**	−2.73 (−5.59–0.13)	−0.81 (−1.65–0.02)	**−0.96 (−1.82–−0.10)**
**Loss of family member/close friend**						
No	Ref	Ref	Ref	Ref	Ref	Ref
Yes	−0.11 (−1.81–1.60)	0.24 (−1.29–1.77)	0.22 (−1.87–2.31)	−0.24 (−2.36–1.89)	0.26 (−0.35–0.86)	0.19 (−0.44–0.83)

Coef: Coefficient, Ref: Reference, CI 95%: Confidence interval at 95%, C. Anxiety: Anxiety score, C. Depression: Depression score, C., and PTSD: Post-Traumatic Stress Disorder. * Adjusted by age, gender, marital status, number of children, profession, working hospital, occupation, working hours, pathological history, and loss of family member/close friend. Estimates in bold had significant *p* values.

## Data Availability

The data that support the findings of this study will be provided upon reasonable request to the corresponding author.
